# Encystment of parasitic freshwater pearl mussel (*Margaritifera margaritifera*) larvae coincides with increased metabolic rate and haematocrit in juvenile brown trout (*Salmo trutta*)

**DOI:** 10.1007/s00436-017-5413-2

**Published:** 2017-03-10

**Authors:** Karl Filipsson, Jeroen Brijs, Joacim Näslund, Niklas Wengström, Marie Adamsson, Libor Závorka, E. Martin Österling, Johan Höjesjö

**Affiliations:** 1 Department of Biological and Environmental Sciences, University of Gothenburg, SE 413 90 Gothenburg, Sweden; 2 Department for Environmental and Life Sciences, Karlstad University, SE 651 88 Karlstad, Sweden; 3 Swedish Anglers Association, Sjölyckan 6, SE 416 55 Gothenburg, Sweden; 4 Laboratoire Évolution & Diversité Biologique (EDB UMR 5174), CNRS, Université de Toulouse, Toulouse, France

**Keywords:** Glochidia, Haematocrit, Host, *Margaritifera*, Metabolic rate, Parasite

## Abstract

Gill parasites on fish are likely to negatively influence their host by inhibiting respiration, oxygen transport capacity and overall fitness. The glochidia larvae of the endangered freshwater pearl mussel (FPM, *Margaritifera margaritifera* (Linnaeus, 1758)) are obligate parasites on the gills of juvenile salmonid fish. We investigated the effects of FPM glochidia encystment on the metabolism and haematology of brown trout (*Salmo trutta* Linnaeus, 1758). Specifically, we measured whole-animal oxygen uptake rates at rest and following an exhaustive exercise protocol using intermittent flow-through respirometry, as well as haematocrit, in infested and uninfested trout. Glochidia encystment significantly affected whole-animal metabolic rate, as infested trout exhibited higher standard and maximum metabolic rates. Furthermore, glochidia-infested trout also had elevated levels of haematocrit. The combination of an increased metabolism and haematocrit in infested fish indicates that glochidia encystment has a physiological effect on the trout, perhaps as a compensatory response to the potential respiratory stress caused by the glochidia. When relating glochidia load to metabolism and haematocrit, fish with low numbers of encysted glochidia were the ones with particularly elevated metabolism and haematocrit. Standard metabolic rate decreased with substantial glochidia loads towards levels similar to those of uninfested fish. This suggests that initial effects visible at low levels of encystment may be countered by additional physiological effects at high loads, e.g. potential changes in energy utilization, and also that high numbers of glochidia may restrict oxygen uptake by the gills.

## Introduction

Parasites can negatively affect their hosts through direct manipulation, inducing immunological responses, and increasing energetic demands, which in many cases influence the behaviour and fitness of the host (Lehmann 1993; Moore 2002). A variety of aquatic parasites, such as the larvae (glochidia) of many species of unionoid mussels, attaches and encysts on the gills of fish (Crane et al. 2011; Denic et al. 2015; Meyers et al. 1980; Young and Williams 1984). Such encystment can affect both the physiology and behaviour of the host. In response to glochidia encystment, fish hosts have been shown to hyperventilate (Crane et al. 2011), undergo haematological alterations (Meyers et al. 1980) and exhibit immunological reactions (Alvarez-Pellitero 2008). The effects of these alterations include impaired gas exchange (Kaiser 2005), increased risk-taking (Godin and Sproul 1988), reduced foraging (Österling et al. 2014) and competitive ability (Filipsson et al. 2016), reduced swimming performance and, in cases of high infestation rates ( ~350 glochidia per gram fish weight or more), even mortality (Taeubert and Geist 2013). For example, glochidia encystment on largemouth bass (*Micropterus salmoides* (Lacepède, 1802)) resulted in higher ventilation rates, reduced oxygen consumption and decreased tolerance of low-oxygen conditions for the host fish, suggesting that gill parasites can pose a respiratory burden for their hosts (Kaiser 2005). In another example, three-spined sticklebacks (*Gasterosteus aculeatus* Linnaeus, 1758) increase their foraging time when infected by parasites even though this poses a greater risk for predation, which suggests that the higher energy demand imposed by the parasites leads to increased risk-taking in order to obtain enough food to sustain fitness (Godin and Sproul 1988).

The freshwater pearl mussel (FPM, *Margaritifera margaritifera* (Linnaeus, 1758)) is an extremely long-lived unionoid bivalve with a Holarctic distribution (Geist 2010; Graf and Cummings 2007; Lopes-Lima et al. 2016). It is endangered throughout its range, yet geographically widely distributed in European streams (Lopes-Lima et al. 2016). In Europe, glochidia of the FPM are obligate parasites on the gills of juvenile brown trout (*Salmo trutta* Linnaeus, 1758) and Atlantic salmon (*Salmo salar* Linnaeus, 1758) (Young and Williams 1984). Where the two species occur in sympatry, both may function as hosts for FPM (Karlsson et al. 2014). In some streams, however, only one of the species is the host for certain FPM populations, even though both brown trout and Atlantic salmon are present (Geist et al. 2006; Ieshko et al. 2016; Karlsson et al. 2014). Large conservation efforts have been made to protect FPM and much research is conducted to understand the causes for its decline and its interactions with the salmonid host species (Geist 2010; Karlsson et al. 2014; Lopes-Lima et al. 2016; Österling and Söderberg 2015). The glochidia are released by gravid females during summer and reach their host passively via the water current. After inhalation by a host fish, glochidia attach and encyst on epithelial cells located on the gill lamellae where they absorb nutrients from the gill tissue (Denic et al. 2015; Nezlin et al. 1994). Glochidia are encysted for approximately 10 months, during which they grow approximately 6–10 times in size whilst metamorphosing to juvenile mussels (Bauer and Vogel 1987; Denic et al. 2015).

Infestation of FPM glochidia in juvenile salmonids has traditionally been assumed to be more or less benign to host fish (Moorkens 1999). However, recent studies reveal that FPM glochidia encystment can increase the recovery time required following a stressful event (Thomas et al. 2014), induce an immune response (Thomas et al. 2014), decrease foraging success (Österling et al. 2014), negatively impact competitive ability (Filipsson et al. 2016) and reduce the capacity for swimming (Taeubert and Geist 2013). Previous studies suggest that parasite infestation can have significant implications for the host. For example, trematode infestation was reported to lower the standard metabolic rate of Arctic charr (*Salvelinus alpinus* Linnaeus, 1758), which was suggested to be due to potential perturbations in energy utilization (Seppänen et al. 2009). Similarly, brown trout infested with high numbers of glochidia took longer to return to their basal rate of ventilation following a stressful event than uninfested fish, which suggests that the glochidia constitute a respiratory burden (Thomas et al. 2014). Unfortunately, whole-animal oxygen uptake was not quantified in Thomas et al. (2014) and therefore it is currently unknown whether or not FPM glochidia affect the metabolism (i.e. standard and maximum metabolic rates) of host fish.

Haematocrit (Hct, % erythrocytes in total blood volume) can be used as an indicator for oxygen transport capacity in an organism as high levels of erythrocytes allow an increased amount of oxygen to be transported in the blood. Typically, vertebrates that are adapted or acclimatized to increased aerobic demands or oxygen-poor environments usually exhibit elevated Hct values (Sherwood et al. 2005). However, low Hct values are not necessarily consistent with low oxygen transport capacity as it can be compensated via increases in cardiac output (Wang et al. 2014). Several fish species, including salmonids, have reduced Hct values when infested with blood- and tissue-consuming parasites (Grimnes and Jakobsen 1996; Jones and Grutter 2005; Nair and Nair 1983; Paperna et al. 1984), explained as an effect of parasites either causing osmotic failure (as a result of exposed lesions) or by blood ingestion (Grimnes and Jakobsen 1996; Jones and Grutter 2005; Nair and Nair 1983). Reduced Hct can affect oxygen transport capacity through anaemia, which may negatively affect foraging success, swimming capacity, activity and overall survival (Jones and Grutter 2005; Pearson and Stevens 1991). However, if parasites affect oxygen uptake or metabolism, Hct could also increase as a compensatory effect to enhance oxygen transport capacity by splenic release of stored blood cells, plasma loss or increased size of erythrocytes (Pearson and Stevens 1991). Only a few attempts have been made to investigate whether FPM glochidia affect haematological parameters in salmonids. Hct was observed to increase in glochidia-infested chinook (*Oncorhynchus tshawytscha* Walbaum, 1792) and coho (*Oncorhynchus kisutch* Walbaum, 1792) salmon (Meyers et al. 1980). However, this trend is not universal, as Thomas et al. (2014) found no effect of parasitic glochidia on the Hct of infested brown trout. Even, a month after infestation, the spleen of infested trout were enlarged (Thomas et al. 2014), which indicates that glochidia may indeed affect the haematology in this host fish species as this organ is involved in immunological activity, haematopoiesis and haemolysis, and can also function as an erythrocyte reservoir (Fänge and Nilsson 1985; Pearson and Stevens 1991).

The overall aim of this paper is to investigate the potential effects of natural levels of FPM glochidia encystment on the metabolism and haematology of wild juvenile brown trout. Specifically, we measured whole-animal oxygen uptake rates at rest and following an exhaustive exercise protocol (as a proxy for standard and maximal metabolic rates), as well as Hct, in infested and uninfested individuals captured from the wild. We hypothesised that glochidia-infested trout would exhibit increased standard metabolic rate (SMR), maximum metabolic rate (MMR) and Hct when compared to uninfested fish, in order to compensate for respiratory stress caused by encysted glochidia.

## Methods

### Experimental animals and holding conditions

Juvenile 1+ brown trout of mixed sexes were collected in April 2015 from the stream Lindåsabäcken in the Häggån catchment, Sweden (57°40.203′ N 13°3.771′ E), using standardized electrofishing (LR-20B, Smith-Root, USA) (Bohlin et al. 1989). The trout were lightly anaesthetized in the field (MS-222, 5 mL L^−1^, Pharmac Ltd., UK) and visually assessed in order to determine whether they were infested with parasitic glochidia. A total of 62 brown trout were caught (35 infested and 27 uninfested). Fork lengths (range, mean ± SE) were 76–146, 116 ± 3 mm for infested trout and 75–152, 114 ± 4 mm for uninfested trout. Wet weights were 4.0–32.2, 16.5 ± 1.2 g for infested trout and 4.7–36.9, 15.7 ± 1.3 g for uninfested trout. Trout were subsequently transported in a well-aerated tank to the University of Gothenburg, Gothenburg, Sweden. Fish were housed in a 120 L aquarium (0.64 m × 0.48 m × 0.4 m). The aquarium was supplied with freshwater from a flow-through filtration system. Photoperiod followed natural day–light cycles and the water temperature was kept constant at 11.0 ± 0.1 °C.

### Respirometry and exhaustive exercise protocol

Fish were fasted for 9 days to allow gut evacuation prior to use in respirometry experiments. Individual fish were then selected randomly and lightly anaesthetized in water containing 0.5 mL L^−1^ 2-phenoxyethanol (Aldrich Chemistry, Germany). Upon loss of equilibrium, individuals were weighed and placed into cylindrical, intermittent flow-through respirometers (volumes of 0.584 or 1.112 L depending on fish size), which were submerged in a reservoir bath containing flow-through, aerated freshwater (11.0 ± 0.1 °C). The fish were left undisturbed in the respirometers for ~21 h while oxygen consumption was measured using best practices in intermittent flow-through respirometry (see Clark et al. 2013). Briefly, water was continuously circulated through each respirometer using an in-line submersible pump within a recirculation loop, and the partial pressure of oxygen in the water in the respirometers was measured continuously at 0.5 Hz using a FireSting O_2_ system (PyroScience, Aachen, Germany) calibrated in accordance with the supplier’s manual. Automated flush pumps refreshed the water in the respirometers for 5 min in every 20-min period, ensuring that oxygen levels in the respirometers always remained above 90% air saturation. Whole-animal oxygen uptake was calculated from the decline in partial pressure of oxygen in the water of the respirometers during each 15-min period between flush cycles. SMR was calculated for each fish as the mean of the lowest 10% of ln-transformed whole-animal oxygen uptake measurements during the ~21-h period where the fish were left undisturbed after respirometer entry (excluding outliers, which were considered to be >2 SD below the mean of the lowest 10% of values).

Following the ~21-h undisturbed period, fish were individually removed from their respirometers and subjected to an exhaustive exercise protocol (Clark et al. 2013) consisting of a 3-min period of manual chasing around a circular tank (diameter 0.6 m, water depth 0.2 m) containing aerated freshwater at 11.0 ± 0.1 °C. All individuals were visibly exhausted by the end of the 3-min exercise period as highlighted by a lack of response to an experimenter tapping the caudal fin. Immediately following the exercise protocol, fish were returned to their individual respirometers (within 10 s), whereupon respirometers were sealed for 5 min and the MMR was taken as the steepest 3-min slope during this time.

### Glochidia load determination

Glochidia load was estimated for each fish directly after they had been in the respirometers. Trout were anaesthetized (0.5 mL L^−1^ 2-phenoxyethanol) and glochidia load was examined by gently lifting the operculum and gill arches using pincers. The number of encysted glochidia was counted as precisely as possible for each fish. However, the number of glochidia on heavily infested fish could not be estimated to an exact number using this method. Thus, trout with more than 100 glochidia encysted on their gills were set to have 100 glochidia when analysing the data.

### Hct measurements

Following glochidia determination, blood samples were obtained for the determination of Hct. By using heparinized 23 G × 31-in. Sterican® single-use hypodermic needles (B. Braun Medical, Emmenbrücke, Switzerland) attached to 1-mL syringes, it was possible to withdraw ~0.1 mL of blood from an individual via puncture of the venous vasculature directly caudal of the anal fin. However, due to the small size of some individuals, it was not possible to withdraw adequate blood samples from all fish, thus a smaller sample size for the determination of Hct was obtained (34 infested and 24 uninfested fish). Hct was determined as the fractional red cell volume upon centrifugation of a subsample of blood in 80-μL microcapillary tubes at 10,000 rpm for 5 min.

### Statistical analyses

Fork length, body mass and condition factor of infested and uninfested fish were compared using independent sample *t* tests. Homoscedasticity and normal distribution of residuals were assessed and found to be appropriate assumptions, by inspection of spread and symmetry of boxplots and Q–Q plots. Relationships between size (fork length) and number of glochidia on the gills were investigated using Spearman rank correlations.

Differences between glochidia-infested and uninfested individuals were analysed using linear models (LMs) including presence/absence of glochidia as a fixed two-level factor and ln (wet mass) as a covariate. Size effects were corrected for in the statistical model, using ln-transformed wet mass as a covariate. Given that metabolic rate is a power function of body mass, we assumed a linear relationship between ln-transformed metabolic rate and ln-transformed wet mass. This was also found to be an appropriate assumption based on inspection of scatterplots. Assumptions regarding residual normality and homoscedasticity of residuals were judged to be adequately fulfilled by visual inspection of residuals (boxplots and Q–Q plots). Differences between infested and uninfested fish were assessed based on the estimated marginal means (EMM) at the mean value of the covariate.

**Fig. 1:**
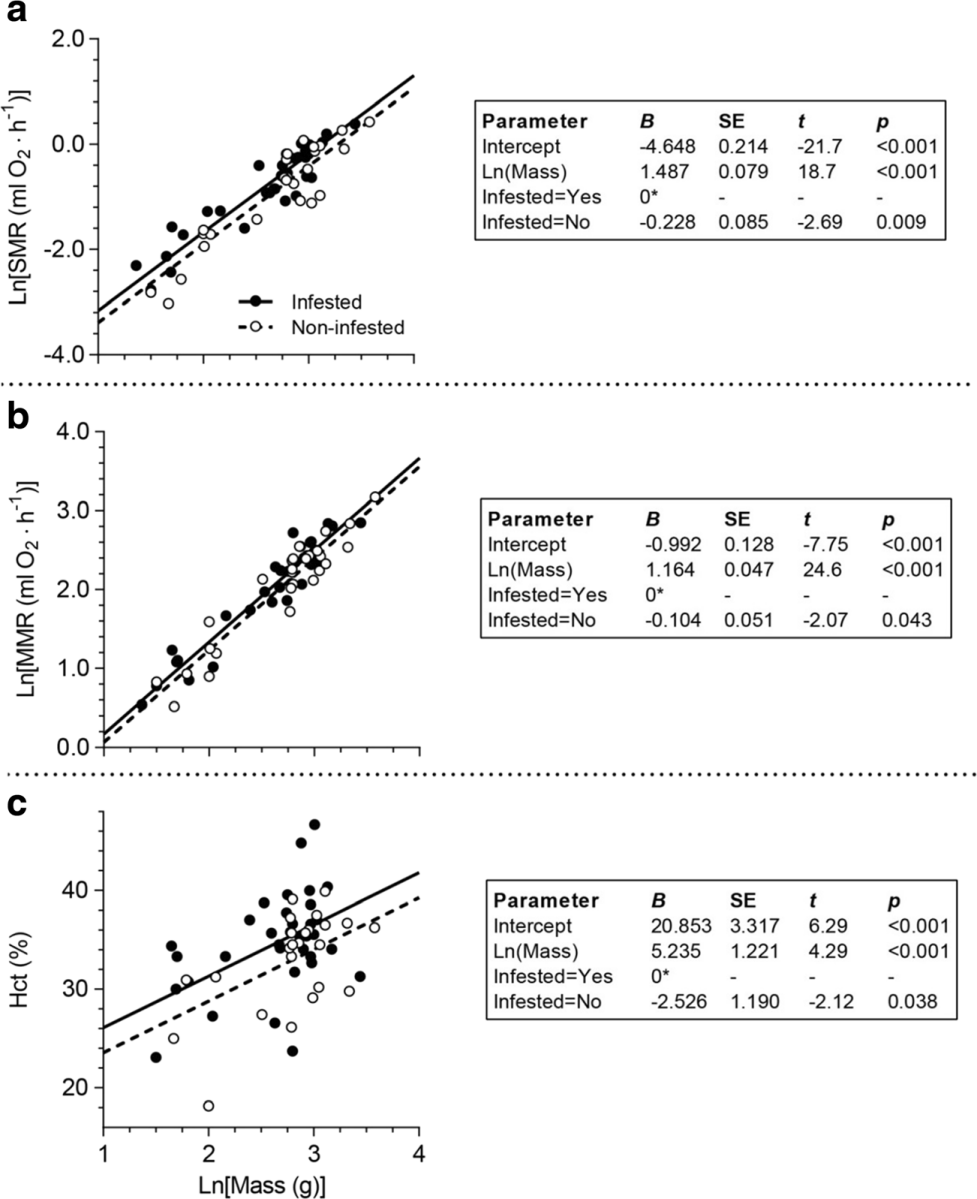
Linear relationships between mass (ln-transformed) and **a** standard metabolic rate (SMR) (ln-transformed), **b** maximum metabolic rate (MMR) (ln-transformed) and **c** haematocrit (Hct), for naturally infested and uninfested brown trout. Parameter estimates (*B*) and their standard error (SE) for the linear regression analysis and their statistical significance (presented as *t* statistics along with their *p* value) are presented in the *boxes* to the right of the graphs, and *asterisks* denote redundant parameter estimate

For infested fish, we descriptively investigated whether glochidia load affected SMR, MMR or Hct, using local regression (LOESS), with glochidia load used as independent variable and Spearman rank correlations. The SMR, MMR and Hct values for each individual were size-standardized as residuals from the LMs used to investigate general effects of infestation (see previous paragraph and Fig. 1).

## Results

### Fish size and condition

There were no significant differences between infested fish and uninfested fish in fork length, body mass or condition factor (all *p* > 0.45). There was no statistically significant rank correlation between size (fork length) and glochidia load (Spearman’s *ρ* = −0.19, *p* = 0.14).

### Effects of glochidia encystment on SMR, MMR and Hct

When controlling SMR for the positive effect of body mass (*F*_1,59_ = 351, *p* < 0.001; Fig. 1a), trout infested with glochidia had a higher SMR than uninfested fish (*F*_1,59_ = 7.26, *p* = 0.009; Fig. 1a). On the arithmetic scale, the infested individuals had on average 26% higher SMR (back-transformed EMM infested = 0.486 mg O_2_·h^−1^, 95% CI: 0.435–0.543 mg O_2_·h^−1^; uninfested = 0.387 mg O_2_·h^−1^, 95% CI: 0.341–0.440 mg O_2_·h^−1^) at the mean of the covariate [ln(mass) = 2.64 (14.0 g)]. Similarly, after controlling for a positive body mass effect (*F*_1,59_ = 603, *p* < 0.001; Fig. 1b), the MMR was higher in infested trout compared to that in uninfested fish (*F*_1,59_ = 4.27, *p* = 0.043; Fig. 1b) with the infested individuals having on average 11% higher MMR (back-transformed EMM infested: 8.01 mg O_2_·h^−1^, 95% CI: 7.50–8.57 mg O_2_·h^−1^; uninfested: 7.22 mg O_2_·h^−1^, 95% CI: 6.69–7.79 mg O_2_·h^−1^) at the mean of the covariate [ln(mass) = 2.64 (14.0 g)]. Hct was also positively related to body size (*F*_1,55_ = 18.4, *p* < 0.001; Fig. 1c) and infested fish had higher Hct than uninfested fish (*F*_1,55_ = 4.50, *p* = 0.038; Fig. 1c). Infested fish had on average Hct that was 2.53% higher than uninfested fish (95% CI of the difference 0.14–4.91 percentage points). Parameter estimates (*B*) for all three linear models are presented in Fig. 1.

### Effects of glochidia load on SMR, MMR and Hct

**Fig. 2:**
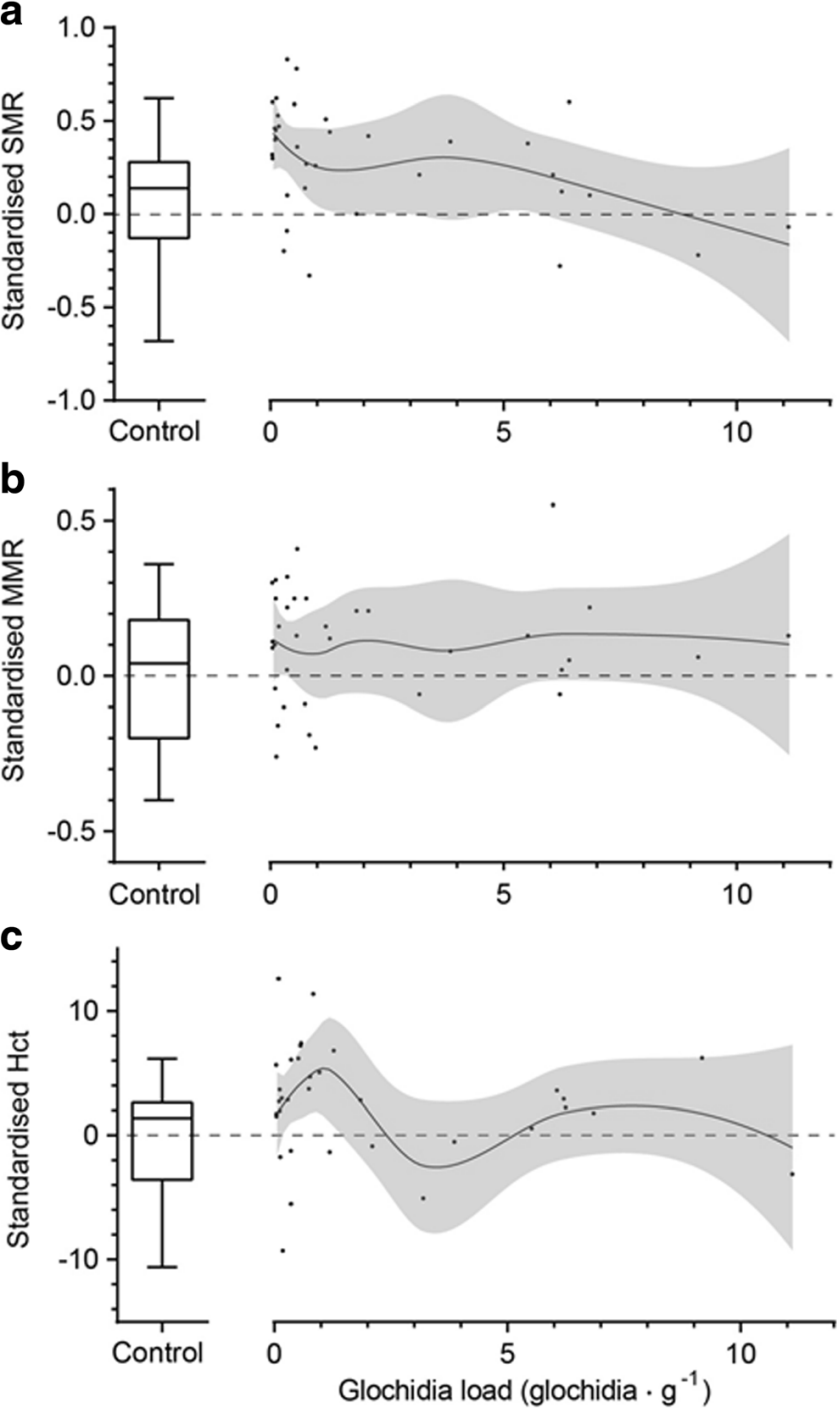
Effects of glochidia load on **a** standard metabolic rate (SMR), **b** maximum metabolic rate (MMR) and **c** haematocrit (Hct). Boxplots on the left-hand side of each figure show the distribution of values for uninfested fish, the *whiskers* span between maximum and minimum values, the *box* delimits the upper and lower quartile, and the *horizontal bar* within the box shows the median. In the right-hand scatterplots, showing data in relation to glochidia load, the *lines* show the local regression (LOESS), with 95% confidence limits in *grey*. All data was standardized for size by using residuals from the respective linear model presented in Fig. 1

We found that size-standardized SMR tended to decrease with increasing glochidia load, which made individuals with high glochidia loads resemble the average uninfested fish in their mean SMR values (Fig. 2a). Rank correlation between SMR and glochidia load was significantly negative (Spearman’s *ρ* = −0.40, *p* = 0.017). No tendencies for effects of glochidia load were found for MMR (*ρ* = −0.04, *p* = 0.81; Fig. 2b) or Hct (*ρ* = −0.08, *p* = 0.66; Fig. 2c).

## Discussion

Glochidia-infested trout exhibited significantly higher SMR and MMR and elevated levels of Hct, which demonstrates the potential of encysted glochidia to affect whole-animal metabolic rate and oxygen transport capacity. Furthermore, glochidia-infested trout also had elevated levels of Hct. The combination of increased metabolism and increased Hct values in infested fish indicates that there is a physiological effect on the trout caused by the glochidia, perhaps as a compensatory response to the potential respiratory stress caused by glochidia encystment.

Similarly, our finding that glochidia-infested trout had higher Hct is similar to that of a previous study, whereby Hct of chinook salmon increased following infestation by western pearl shell (*Margaritifera falcata* Gould, 1850) glochidia (Meyers et al. 1980). The increased Hct of infested chinook salmon was related to a combination of increased number of circulating erythrocytes and cell swelling (i.e. increased mean corpuscular volume and reduced mean corpuscular haemoglobin concentration) (Meyers et al. 1980). This may also be the case for the infested brown trout in the present study, however, future measures of cell number and size are necessary to substantiate these claims.

When relating glochidia load to metabolism and Hct, the fish with lower numbers of glochidia per gram body mass were the ones with particularly elevated metabolism (SMR) and Hct. Interestingly, SMR then decreased with substantial glochidia loads towards levels similar to those of uninfested fish. This is not necessarily suggesting that trout with high glochidia loads are unaffected. Rather, we hypothesise that the initial effects visible at low levels of encystment may be countered by additional physiological effects at high loads (e.g. potential perturbations in energy utilization as suggested by Seppänen et al. 2009). Previous studies have shown that low glochidia loads (<100 glochidia per gram body mass) are not lethal and do not necessarily affect the host salmonid performance, at least when kept under aquaculture conditions (Taeubert and Geist 2013; Treasurer et al. 2006). However, very high glochidia loads have been shown to cause mortality in brown trout (up to 60% mortality within 48 h post-infestation with loads of ~900 glochidia per gram body mass) (Taeubert and Geist 2013). Furthermore, swimming performance decreases with increasing glochidia load, which could be an effect of increased oxygen demand for a given activity or reduced capacity to take up oxygen due to damage on gills in heavily infested fish (Taeubert and Geist 2013).

After infestation, glochidia are fully enveloped in the gill tissue, typically the filament (Karna and Millemann 1978), as a consequence of shape change and migration of gill epithelial cells, without signs of hyperplasia (Nezlin et al. 1994). After a few days following encystment, the ultrastructure of cysts and normal gill tissue is very similar (Nezlin et al. 1994). However, filaments with encysted glochidia are on average thicker and longer than normal filaments (Thomas et al. 2014). The lamellae of the filaments often fuse with the wall of the cyst, and while blood still flows through these lamellae, they may not function to the same extent for respiration (Karna and Millemann 1978). Glochidia can also pinch arterioles, restricting blood flow to certain filaments, and large cysts increase the physiological dead space in the water flow across the gills (Karna and Millemann 1978). Our results, where basal oxygen consumption first increases at low glochidia loads and then decreases again with increasing load, indicates that higher numbers of glochidia may restrict oxygen uptake by the gills and a mechanism for this effect may be restriction of blood flow or respiratory functional surface.

Indeed, previous data show that brown trout infested with high numbers of glochidia take longer to reach basal ventilation rates after a stressor, suggesting that high glochidia loads indeed constitute a respiratory burden (Thomas et al. 2014). If gill parasites reduce oxygen uptake from the gills, an adaptive response to parasite attachment could be to increase the capacity of the blood to carry oxygen, e.g. through increasing the number of erythrocytes or haemoglobin (Meyers et al. 1980). Although there are indications that this may be occurring in the present study (i.e. significantly higher Hct in infested brown trout), a more extensive haematological evaluation of infested and uninfested fish is necessary to validate this hypothesis.

A word of caution is warranted as whole-animal oxygen uptake and Hct may reflect pre-existing differences in the trout phenotypes with infested fish having a generally higher metabolism and activity, which in turn may have caused an increased exposure to glochidia in the wild (Wengström et al. 2016). Therefore, the effect of glochidia on oxygen uptake capacity should be further investigated in future studies, using artificially infested fish with natural levels of glochidia encystment. However, our findings still reflect the physiological status of infested and uninfested fish within the stream ecosystem, and when used in combination with results from the previous studies outlined above (Meyers et al. 1980; Thomas et al. 2014), it seems likely that the effects seen in our study are indeed caused by glochidia encystment.

The natural glochidia loads in this study are notably lower than the loads where adverse effects are noted in previous studies. Juvenile brown trout examined in the present study were captured approximately 7–9 months after initial glochidia infestation, compared to e.g. 48-h post-infestation as the fish studied by Taeubert and Geist (2013). It is likely that heavily infested fish have been outcompeted or suffered high mortality rates during a longer time period (Filipsson et al. 2016; Taeubert and Geist 2013), explaining the relatively low number of encysted glochidia found on the fish in this study. Additionally, many glochidia are sloughed off the gills during the initial period of encystment, as the trout’s immune system responds to glochidia infection (Hastie and Young 2003). Hence, this may also lead to a decrease in encysted glochidia in host fish populations over time.

When artificially infesting trout with glochidia in mussel conservation programmes, the recommended load on the host fish is between 5 and 100 glochidia per gram body mass (Taeubert and Geist 2013). The fish in our study are well within this recommended span, and we therefore note, with support from other studies on behaviour (Filipsson et al. 2016; Österling et al. 2014; Wengström et al. 2016), that the higher loads within this span may also be affecting the host fish negatively, to the potential detriment of the mussel glochidia. Based on our findings, we reiterate the conclusion that artificial glochidia infestation of fish within pearl mussel propagation programmes should be done with care and aim for relatively low glochidia encystment rates. This could avoid detrimental effects on host fish, which may translate into unsuccessful deployment of juvenile mussels into the stream where the infested fish are released (Taeubert and Geist 2013; Thomas et al. 2014).

## References

[CR1] Alvarez-Pellitero P (2008). Fish immunity and parasite infections: from innate immunity to immunoprophylactic prospects. Vet Immunol Immunopathol.

[CR2] Bauer G, Vogel C (1987) The parasitic stage of the freshwater pearl mussel (*Margaritifera margaritifera* L.). I. Host response to glochidiosis. Arch Hydrobiol 76:393–402

[CR3] Bohlin T, Hamrin S, Heggberget TG, Rasmussen G, Saltveit SJ (1989) Electrofishing - Theory and practice with special emphasis on salmonids. Hydrobiologia 173:9–43

[CR4] Clark TD, Sandblom E, Jutfelt F (2013). Aerobic scope measurements of fishes in an era of climate change: respirometry, relevance and recommendations. J Exp Biol.

[CR5] Crane AL, Fritts AK, Mathis A, Lisek JC, Barnhart MC (2011). Do gill parasites influence the foraging and antipredator behavior of rainbow darters, *Etheostoma caeruleum*?. Anim Behav.

[CR6] Denic M, Taeubert JE, Geist J (2015). Trophic relationships between the larvae of two freshwater mussels and their fish hosts. Invertebr Biol.

[CR7] Fänge R, Nilsson S (1985). The fish spleen: structure and function. Experientia.

[CR8] Filipsson K, Petersson T, Höjesjö J, Piccolo JJ, Näslund J, Wengström N, Österling EM (2016). Heavy loads of parasitic freshwater pearl mussel (*Margaritifera margaritifera* L.) larvae impair foraging, activity and dominance performance in juvenile brown trout (*Salmo trutta* L.). Ecol Freshw Fish.

[CR9] Geist J (2010). Strategies for the conservation of endangered freshwater pearl mussels (*Margaritifera margaritifera* L.): a synthesis of conservation genetics and ecology. Hydrobiologia.

[CR10] Geist J, Porkka M, Kuehn R (2006). The status of host fish populations and fish species richness in European freshwater pearl mussel (*Margaritifera margaritifera*) streams. Aquatic Conserv: Mar Freshw Ecosyst.

[CR11] Godin J-GJ, Sproul CD (1988). Risk taking in parasitized sticklebacks under threat of predation: effects of energetic need and food availability. Can J Zoolog.

[CR12] Graf DL, Cummings KS (2007). Review of the systematics and global diversity of freshwater mussel species (Bivalvia: Unionoida). J Molluscan Stud.

[CR13] Grimnes A, Jakobsen PJ (1996). The physiological effects of salmon lice infection on post-smolt of Atlantic salmon. J Fish Biol.

[CR14] Hastie LC, Young MR (2003) Conservation of the freshwater pearl mussel—1. Captive breeding techniques (IN127). Conserving Natura 2000 Rivers Conservation Techniques Series No. 2. English Nature, Peterborough

[CR15] Ieshko EP, Geist J, Murzina SA, Veselov AE, Lebedeva DI, Ziuganov VV (2016). The characteristics of the infection of juvenile Atlantic salmon with glochidia of the freshwater pearl mussel in rivers of Northwest Russia. Knowl Manag Aquat Ecosyst.

[CR16] Jones CM, Grutter AS (2005). Parasitic isopods (*Gnathia* sp.) reduce haematocrit in captive blackeye thicklip (Labridae) on the Great Barrier Reef. J Fish Biol.

[CR17] Kaiser BE (2005) The effects of glochidiosis on fish respiration. Unpublished MSc thesis, Missouri State University, Springfield, MO

[CR18] Karlsson S, Larsen BM, Hindar K (2014). Host-dependent genetic variation in freshwater pearl mussel (*Margaritifera margaritifera* L.). Hydrobiologia.

[CR19] Karna D, Millemann RE (1978). Glochidiosis of salmonid fishes. III. Comparative susceptibility to natural infection with *Margaritifera margaritifera* (Pelecypoda: Magaritanidae) and associated histopathology. J Parasitol.

[CR20] Lehmann T (1993). Ectoparasites: direct impact on host fitness. Parasitol Today.

[CR21] Lopes-Lima M (2016). Conservation status of freshwater mussels in Europe: state of the art and future challenges. Biol Rev.

[CR22] Meyers TR, Millemann RE, Fustish CA (1980). Glochidiosis of salmonid fishes. IV. Humoral and tissue responses of coho and chinook salmon to experimental infection with *Margaritifera margaritifera* (L.) (Pelecypoda: Margaritanidae)*. J Parasitol.

[CR23] Moore J (2002). Parasites and the behavior of animals.

[CR24] Moorkens EA (1999) Conservation management of the freshwater pearl mussel *Margaritifera margaritifera*. Part 1: biology of the species and its present situation in Ireland. Irish Wildlife Manuals, No 8 Dublin: Dúchas, The Heritage Service

[CR25] Nair GA, Nair NB (1983). Effect of infestation with the isopod, *Alitropus typus* M. Edwards (Crustacea: Flabellifera: Aegidae) on the haematological parameters of the host fish *Channa striatus* (Bloch). Aquaculture.

[CR26] Nezlin LP, Cunjak RA, Zotin AA, Ziuganov VV (1994). Glochidium morphology of the freshwater pearl mussel (*Margaritifera margaritifera*) and glochidiosis of Atlantic salmon (*Salmo salar*): a study by scanning electron microscopy. Can J Zoolog.

[CR27] Österling EM, Söderberg H (2015). Sea-trout habitat fragmentation affects threatened freshwater pearl mussel. Biol Conserv.

[CR28] Österling EM, Ferm J, Piccolo JJ (2014). Parasitic freshwater pearl mussel larvae (*Margaritifera margaritifera* L.) reduce the drift-feeding rate of juvenile brown trout (*Salmo trutta* L.). Environ Biol Fish.

[CR29] Paperna I, Diamant A, Overstreet RM (1984). Monogenean infestations and mortality in wild and cultured Red Sea fishes. Helgolander Meeresunters.

[CR30] Pearson MP, Stevens ED (1991). Splenectomy impairs swim performance in trout. Can J Zoolog.

[CR31] Seppänen E, Kuukka H, Voutilainen A, Huuskonen H, Peuhkuri N (2009). Metabolic depression and spleen and liver enlargement in juvenile Arctic charr *Salvelinus alpinus* exposed to chronic parasite infection. J Fish Biol.

[CR32] Sherwood L, Klandorf H, Yancey P (2005). Animal physiology: from genes to organisms.

[CR33] Taeubert JE, Geist J (2013). Critical swimming speed of brown trout (*Salmo trutta*) infested with freshwater pearl mussel (*Margaritifera margaritifera*) glochidia and implications for artificial breeding of an endangered mussel species. Parasitol Res.

[CR34] Thomas GR, Taylor J, Garcia de Leaniz C (2014). Does the parasitic freshwater pearl mussel *M. margaritifera* harm its host?. Hydrobiologia.

[CR35] Treasurer JW, Hastie LC, Hunter D, Duncan F, Treasurer CM (2006). Effects of (*Margaritifera margaritifera*) glochidial infection on performance of tank-reared Atlantic salmon (*Salmo salar*). Aquaculture.

[CR36] Wang T, Lefevre S, Iversen NK, Findorf I, Buchanan R, McKenzie DJ (2014). Anaemia only causes a small reduction in the upper critical temperature of sea bass: is oxygen delivery the limiting factor for tolerance of acute warming in fishes?. J Exp Biol.

[CR37] Wengström N, Wahlqvist F, Näslund J, Aldvén D, Závorka L, Österling EM, Höjesjö J (2016). Do individual activity patterns of brown trout (*Salmo trutta*) alter the exposure to parasitic freshwater pearl mussel (*Margaritifera margaritifera*) larvae?. Ethol.

[CR38] Young MR, Williams J (1984). The reproductive biology of the freshwater pearl mussel *Margaritifera margaritifera* (Linn.) in Scotland 1. Field studies. Arch Hydrobiol.

